# Better Alone Than in Bad Company: Trophic Ecology of Co‐Occurring Invasive and Native Crayfish

**DOI:** 10.1002/ece3.71385

**Published:** 2025-05-14

**Authors:** Daniela Ghia, Gianluca Fea, Annagiulia Murtas, Martina Ventimiglia, Tiziano Bo, Andrea Basso, Tobia Pretto, Roberto Sacchi, Fabio Ercoli

**Affiliations:** ^1^ Dipartimento di Scienze Della Terra e Dell'Ambiente Università Degli Studi di Pavia Pavia Italy; ^2^ Chair of Hydrobiology and Fishery Estonian University of Life Sciences, Institute of Agricultural and Environmental Sciences Tartu Estonia; ^3^ Dipartimento di Scienze Della Vita e Biologia Dei Sistemi Università Degli Studi di Torino Torino Italy; ^4^ Istituto Zooprofilattico Sperimentale Delle Venezie Centro Specialistico Ittico Legnaro Italy; ^5^ Department of Biological and Environmental Sciences University of Jyväskylä Jyväskylä Finland

**Keywords:** *Austropotamobius pallipes*, condition indices, diet, *Pacifastacus leniusculus*, stable isotopes, stomach content, sympatric species, trophic niche

## Abstract

The North American signal crayfish, *Pacifastacus leniusculus* , is one of the most successful invasive crayfish species in Europe. Its broad trophic niche and ability to exploit various food sources across different trophic levels, coupled with the spread of lethal crayfish disease, pose significant threats to native crayfish populations. However, documentation of co‐occurrence between invasive signal crayfish and native crayfish in invaded freshwater ecosystems remains rare, and research on their coexistence remains limited. In an Italian stream, signal crayfish coexist with native, white‐clawed crayfish, *Austropotamobius pallipes* . This study investigated the trophic ecology of signal crayfish and white‐clawed crayfish at sites where they co‐occurred versus those where they occurred alone. We evaluated whether ecological traits, such as trophic niche, the presence of crayfish plague ( *Aphanomyces astaci* ), and body condition of signal crayfish at the invasion front, facilitated the invasion progress of signal crayfish and replacement of native white‐clawed crayfish. The research employed stable isotope analyses of carbon and nitrogen, using SIBER and MixSIAR mixing models, along with stomach content analyses and Fulton and hepatopancreas indices. When the two species occurred alone, they exhibited trophic niche partitioning. When they coexisted, their trophic niches significantly overlapped. Specifically, signal crayfish shifted their trophic niche to that of white‐clawed crayfish, changing from a predatory‐omnivorous diet to a primary consumer. A greater occurrence of crayfish was found in the stomachs of signal crayfish compared to white‐clawed crayfish, indicating higher cannibalistic behaviour, while both species consumed substantial proportions of macroinvertebrates, detritus, and periphyton when co‐occurring. In general, signal crayfish exhibited better conditions when co‐occurring with native species compared to allopatric individuals, suggesting higher strength in individuals at the invasion front. This study highlights the complex dynamics of invasive and native crayfish interactions, emphasising the greater trophic plasticity and improved biological conditions exhibited by invasive signal crayfish during co‐occurrence.

## Graphical abstract

This study investigated and compared trophic interactions between invasive signal crayfish and native white‐clawed crayfish at sites of co‐occurrence and in allopatry. The presence of crayfish plague, body condition, and trophic niche traits were explored as possible factors that make invasive signal crayfish a strong competitor, enhancing its expansion and replacing the native white‐clawed crayfish. In the absence of crayfish plague, this study emphasises the greater trophic plasticity and biological conditions exhibited by invasive signal crayfish during co‐occurrence.

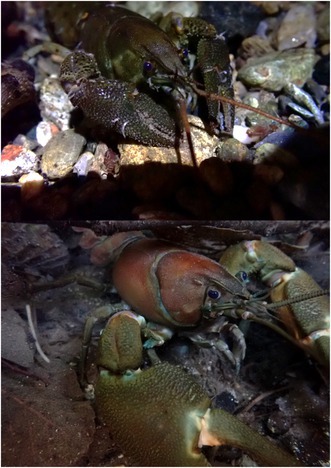

## 1 Introduction

The study of aquatic ecosystems often reveals multiple interactions between native and invasive species (Tran et al. 2015; Taskinen et al. 2021; Dominguez Almela et al. 2021). Interactions between invasive and native species are complex and multifaceted, which have significant implications for biodiversity conservation and ecosystem functioning. Biological invasions, where non‐native species are introduced and proliferate in new environments, pose a pervasive and costly environmental challenge, leading to extensive research and management efforts (Kennedy et al. 2002; Simberloff et al. 2013; Guareschi et al. 2024).

Invasive species can have detrimental effects on native communities (Dudgeon et al. 2006; Ercoli et al. 2015; Roy et al. 2024) and alter essential ecological processes, such as modifying trophic webs and disrupting energy fluxes (Bo et al. 2012; Ercoli et al. 2024). Understanding these interactions is crucial for developing strategies to mitigate the negative impacts of biological invasions and protect native habitats. Invasive species typically display a wider trophic niche than natives (Olsson et al. 2009; Candiotto et al. 2011; Ercoli et al. 2014; Yalçın Özdilek et al. 2019; Modesto et al. 2021), indicating higher trophic plasticity and adaptability to newly invaded ecosystems, which makes them strong competitors.

In some cases, invasive and native species can coexist while also exhibiting ecological strategies, such as partitioning or shifting of trophic niches (Zwerschke et al. 2018; Pacioglu et al. 2020; Viana et al. 2023; Ercoli et al. 2024). When ecologically similar species share the same habitat, competition can alter their trophic niche width (Ercoli et al. 2025). These changes may result in species becoming more specialised as the range of resources they rely on to avoid direct competition becomes more depleted. Conversely, individuals forced to exploit alternative food sources or habitat, due to increased competition for resources, may adopt a more generalist diet (Jackson et al. 2012, 2016; Laiolo et al. 2017).

The fitness and general condition of freshwater invasive species on the invasion front are crucial for successful population establishment (Rahel and Olden 2008). At the invasion front, individuals encounter new environmental conditions, such as changes in water quality, temperature, and food availability, as well as competition with native species. Successful invasive species at the invasion front are typically more ecologically adaptable, displaying more dominant and aggressive behaviour (Lele and Pârvulescu 2017) and having better body condition compared to core populations (Hudina et al. 2015). These traits provide a reproductive advantage, higher survival, and stronger competitive abilities over the native counterpart. Successful invasive species in their invasion front tend to be in excellent condition, with an enhanced ability to propagate further into the host ecosystems (García‐Berthou 2007).

However, invasion strategies and success by invasive species depend on context and species‐specific life history traits (García‐Berthou 2007; Chucholl and Chucholl 2021; Catford et al. 2022). Further research is needed across different geographical and ecosystem scales to better understand invasion dynamics and the effects and conditions under which co‐occurring invasive and native species compete. By exploring these interactions, valuable insights can be gained into invasion dynamics, aiding the development of more effective conservation and mitigation strategies to preserve the delicate balance of native ecosystems.

**FIGURE 1:**
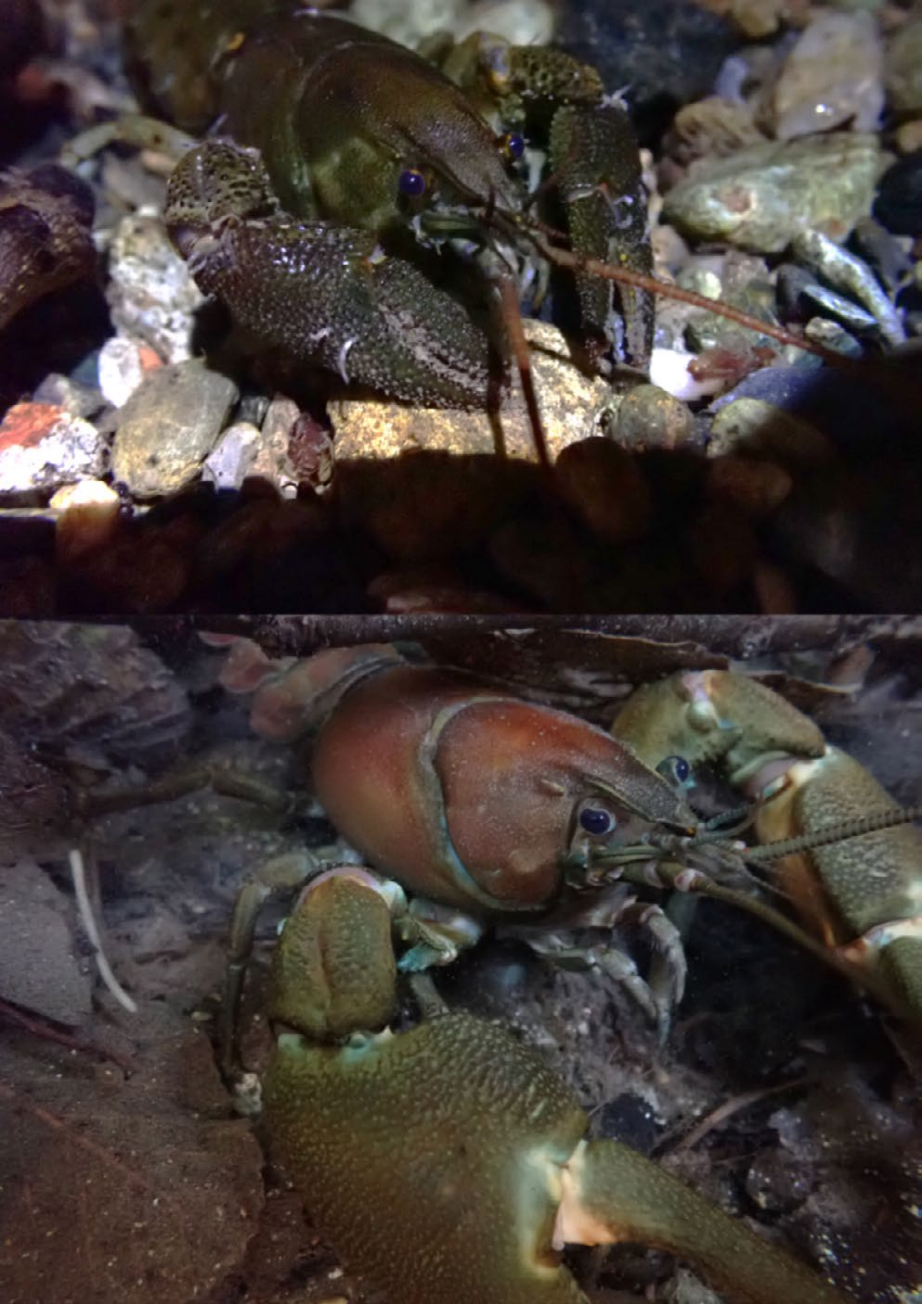
The native white clawed crayfish (above) and the invasive alien signal crayfish (below) photographed in Valla Stream during night sampling and collecting. Both individuals were adult males.

In this research, we investigated the rare co‐occurrence of the invasive signal crayfish *Pacifastacus leniusculus* (Dana, 1852) and the native white‐clawed crayfish *Austropotamobius pallipes* (Lereboullet, 1858) in an Italian stream over a 2‐year period (Figure 1). This rare instance of co‐occurrence presents a unique opportunity to understand the interactions between these species, which typically do not coexist due to competitive pressures exerted by invasive signal crayfish and its ability to transmit the lethal crayfish plague (*Aphanomyces astaci*, Schikora 1906) to native crayfish (Robinson et al. 2018; Svoboda et al. 2017).

Understanding the dynamics that enable invasion progress by signal crayfish, at the expense of native, white‐clawed crayfish, will provide valuable insights into mitigating the impacts of invasive species and enhancing the resilience of native populations.

The objectives of this study were to: (i) investigate and compare the trophic interactions between signal crayfish and white‐clawed crayfish when co‐occurring (sympatry) and when solely inhabiting (allopatry) the same stream; and (ii) identify potential factors facilitating the invasion of signal crayfish at the expense of native crayfish.

We hypothesised that (i) signal crayfish would have wider trophic niches in sympatry than in allopatry due to their ability to exploit a wider trophic range of resources, while occupying a similar trophic niche as white‐clawed crayfish; (ii) signal crayfish would exhibit better body condition in sympatry than in allopatry due to their ability to exploit wider trophic resources and stronger adaptability behaviour; and (iii) signal crayfish in the study stream would not carry *A. astaci* , since there is the presence of a native species that is highly susceptible to the crayfish plague.

This study aimed to better understand the invasion strategies of signal crayfish and prompt conservation actions aimed at preventing the decline of the endangered native white‐clawed crayfish.

## 2 Material and Methods

### 2.1 Study Area

**FIGURE 2:**
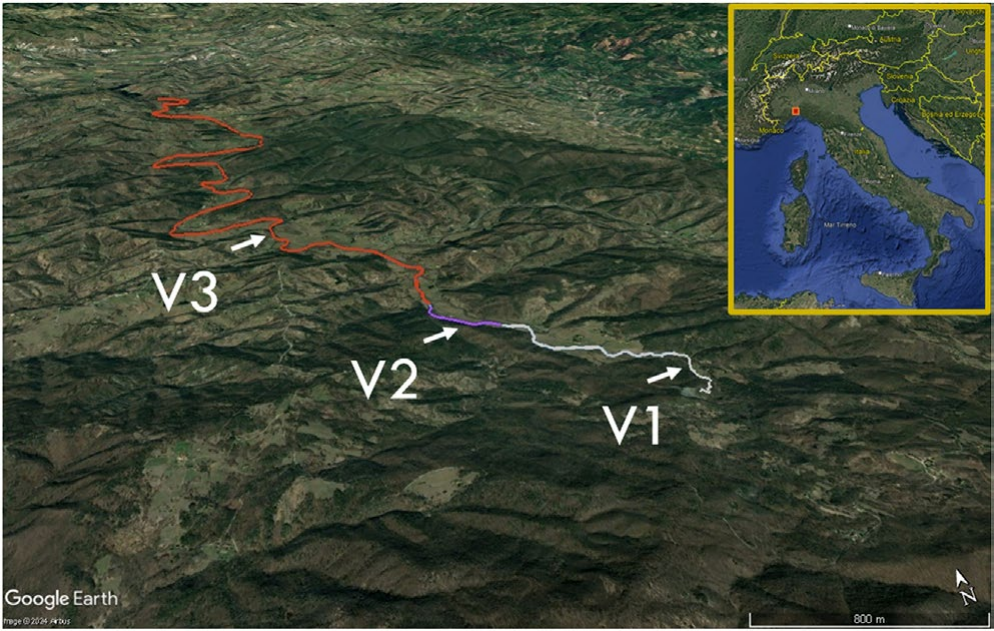
Map of Valla Stream, flowing south–north, and sampling sites. Stream section inhabited by white‐clawed crayfish in allopatry is grey‐coloured (V1), stream section inhabited by both white‐clawed and signal crayfish in sympatry is purple‐coloured (V2), and stream section inhabited by signal crayfish in allopatry is brick red‐coloured (V3).

The study was conducted in Valla Stream, an Apennine stream located in northwest Italy within the Po River basin (Figure 2). The stream flows in a south–north direction for 24 km, ranging from 833 to 222 m above sea level, with an average slope of 2.2%. Valla Stream represents a typical third‐order Apennine lotic environment, characterised by a width ranging from 3.0 to 5.0 m. During summer, the lower section of the stream tends to dry out, leaving isolated pools. Riparian vegetation predominantly consists of *Alnus glutinosa* , *Salix* spp., *Populus* spp., and, occasionally, *Robinia pseudoacacia* . Surrounding land use consists mostly of oak and hornbeam forests, as well as fields, pastures, and scattered houses. Substrate composition of Valla Stream includes coarse‐grained, arenaceous, and conglomeratic successions, with clayey‐arenaceous strata.

### 2.2 Study Species

The white‐clawed crayfish is listed as an endangered species on the IUCN red list (Füreder et al. 2010). Once widely spread, it has undergone a drastic decline in recent decades due to habitat loss and degradation, pollution, water resource exploitation, and the spread of alien species (Manenti et al. 2019). The white‐clawed crayfish can inhabit different types of water bodies, from fast‐moving rivers to canals and lentic waters such as lakes (Souty‐Grosset et al. 2006). However, nowadays, the populations are often limited to hill and foothill streams, where human activity is minimal and where invasive crayfish have not yet been introduced (Ghia et al. 2013; Vezza et al. 2016). A small relict population of white‐clawed crayfish is still present in the headwaters of Valla Stream (Ghia et al. 2019), following a 15‐year decline in the native crayfish population that had previously been present throughout the entire stream (Nardi et al. 2005).

Signal crayfish is one of the most widespread and successful invasive crayfish in Europe, but its presence in Italian freshwater ecosystems is reported for only a few locations (Kouba et al. 2014). Signal crayfish can occupy a wide range of habitats ranging from small streams to large rivers and lakes, such as Lake Tahoe in its original distribution area (Souty‐Grosset et al. 2006). After the first reports of this species in Valla Stream, a thorough study into its trophic ecology was conducted in the downstream section, where the population has been established for a long time (Ercoli et al. 2021). Both species belong to the family Astacidae and exhibit similar characteristics in their reproductive cycles (Yazicioglu et al. 2016).

### 2.3 Sample Collection

For this study, three sites (200 m stretch) were chosen and sampled for 2 consecutive years (2017 and 2018) during summer and early autumn based on preliminary night samplings (Figure 2, Table 1) and distribution of the two species along the stream (Falasco et al. 2018; Ghia et al. 2019). The three sampled sites were similar in terms of habitat types, riparian buffer zones as well as shelters and food availability.

**TABLE 1:** Catch per unit effort (CPUE) values (number (n) of crayfish/min/person). WCC—White‐clawed crayfish, SGC—Signal crayfish.

Site	Species	2017		2018	
		July	October	July	October
V1	WCC	0.37	0.36	0.38	0.67
V2	WCC	0.44	0	0	0.04
V2	SGC	0.04	0	0.14	0.14
V3	SGC	4.0	0.68	3.5	0.73

Summer droughts in some stream reaches did not allow the presence of the sympatric zone (V2) in autumn 2017. Consequently, in October 2017, crayfish were collected from two stretches where the two species were in allopatry (white‐clawed crayfish from site V1 upstream and signal crayfish from site V3 downstream). In October 2018, crayfish were collected from the same two allopatric stretches, as well as from the co‐occurrence zone (V2). In both years, all crayfish were caught by hand at night, when they are more active. For stomach contents and stable isotopes analyses, all crayfish were killed by hypothermia in accordance with European and Italian laws on animal use in scientific research (Tricarico and Zanetti 2023). Due to its endangered status, we were allowed to collect only a few white‐clawed individuals (no more than 30 individuals per year); however, a reasonable number for stable isotope analyses (Jackson and Britton 2014) and stomach content analyses. Sampling and sacrificing of individuals were authorised by Regione Liguria—Dip. Agricoltura, Turismo, Formazione e Lavoro, Parchi e Biodiversità (Aut. Prot. NP/21285, 11/10/2017 and Decreto n.768, 13/08/2018).

From each site in both years, three replicate samples of macroinvertebrates, detritus (coarse particulate organic matter; CPOM), periphyton, and macrophytes were collected as putative food items. Detritus (CPOM) and periphyton represented terrestrial allochthonous oak ( *Quercus robur* ), alder ( *Alnus glutinosa* ), willow (*Salix* spp.), poplar (*Populus* spp.), and black locust ( *Robinia pseudoacacia* ), as well as in‐stream primary production. Macroinvertebrates were sampled semi‐quantitatively by kick‐net, while macrophytes and detritus were collected by hand. Periphyton samples were collected by gently brushing stone surfaces found along the stream bed. All samples were kept cool in the field and frozen after return to the laboratory within a few hours of collection. In the laboratory, macroinvertebrate samples were identified to the lowest feasible taxonomic level (mostly to genus or family) and counted (Campaioli et al. 1994, 1999; Tachet et al. 2002).

Crayfish were sexed, weighed (accuracy ±0.1 g), and measured to record the cephalothorax length (from the tip of the rostrum to the posterior median edge of the cephalothorax) with a digital calliper (accuracy ±0.1 mm). A sample of untreated abdominal muscle tissue from each crayfish was utilised to measure stable isotope ratios (Stenroth et al. 2006). Each crayfish analysed for stable isotopes was also examined for stomach content analyses.

### 2.4 *Aphanomyces astaci* Analyses

The presence of *A. astaci* , the aetiological agent of crayfish plague, was evaluated by molecular analyses for both crayfish species at two different time points. In 2018, 12 signal crayfish, including one from the sympatry site, and two white‐clawed crayfish from the same site (V2), were collected. The specimens were euthanized, and selected portions of their cuticle were collected and stored frozen until molecular analysis (Oidtmann et al. 2006). In 2021, using a non‐invasive protocol with disposable swabs (Basso et al. 2023), 30 signal crayfish and 30 white‐clawed crayfish were collected from the sympatric site (that shifted upstream in the meantime) and swabs were fixed in absolute ethanol until molecular analysis. DNA extractions were performed on both cuticle segments (14 samples) and swabs (60 samples) using the QIAamp DNA Mini Kit (Qiagen GmbH, Hilden, Germany), following the manufacturer's protocol. The excess of fixative was removed by drying the swabs prior to extraction. Analyses were performed using real‐time PCR, applying the primers and probe designed by Vrålstad et al. (2009), with modifications in the thermal protocol according to Strand (2013).

### 2.5 Stable Isotope Analysis (SIA)

Crayfish, macroinvertebrates, periphyton, macrophytes, and detritus samples were dried at 60°C for 48 h and then ground into a fine, homogeneous powder. For animal and plant samples, around 0.6 and 1.0 mg of material were weighed into tin capsules, respectively. Carbon and nitrogen stable isotopes were analysed using a FlashEA1112 elemental analyser connected to a Thermo Finnigan DELTAplus Advantage continuous flow isotope ratio mass spectrometer (Thermo Electron Corporation, Waltham, MA, USA) at the University of Jyväskylä, Finland. Internal standards with known relationships to international standards (Vienna Pee Dee Belemnite for carbon, and atmospheric nitrogen for nitrogen) were used. Carbon and nitrogen stable isotope ratios were reported as parts per thousand (‰) delta values (δ) relative to the international standards. To ensure precision, white muscle tissue from northern pike ( *Esox lucius* ) and birch leaves ( *Betula pendula* ) with known isotopic compositions were used as internal working standards. One standard sample was analysed after every five samples in each sequence. Standard deviations within reference samples were < 0.1‰ for carbon and 0.2‰ for nitrogen in both pike and birch leaf samples.

### 2.6 Trophic Niches and Diets of Crayfish Species

Either carbon or nitrogen stable isotope values, or both of basal resources, such as periphyton (*p* = 0.02; *p* < 0.001), macroinvertebrates (*p* = 0.08; *p* < 0.001), and detritus (*p* = 0.05; *p* = 0.5) varied significantly between sites. To compare trophic niches of signal and white‐clawed crayfish between sites, carbon and nitrogen isotopes of both crayfish species were corrected using the following formulae. For carbon:

$$\delta^{13}C_{\mathrm{cor}}=\left(\delta^{13}C_{i}-\delta^{13}C_{\text{cons}}\right)/\text{range}\left(\delta^{13}C_{\text{cons}}\right)$$

where δ^13^C_i_ were carbon isotope values from an individual crayfish *i*, δ^13^C_cons_ was the mean δ^13^C value of macroinvertebrates from each site, range (δ^13^C_cons_) was the difference between maximum and minimum δ^13^C values of primary consumers (Ephemeroptera and Chironomidae) from each site. For nitrogen:

$$\delta^{15}N_{\mathrm{cor}}=\left(\left(\delta^{15}N_{i}\;–\;\delta^{15}N_{\mathrm{baseline}}\right)/\text{TDF}\right)+2$$

where δ^15^N_i_ were nitrogen isotope values from an individual crayfish *i*, δ^15^N_baseline_ was the mean δ^15^N value of primary consumers (Ephemeroptera and Chironomidae) from each site, and TDF (Trophic Discrimination Factor) was set at 2.6 (Veselý et al. 2024). The trophic level of primary consumers (Ephemeroptera and Chironomidae) selected for the baseline is 2.

Crayfish were categorized by the sampling site (allopatric and sympatric) and size (juveniles and adults) setting the threshold of cephalothorax length (CL) at 28 mm for white‐clawed crayfish and 30 mm for signal crayfish (Ghia et al. 2015; Souty‐Grosset et al. 2006).

The trophic niche width of signal crayfish and white‐clawed crayfish, in allopatry and sympatry, was calculated using the Bayesian Standard Ellipse Area (SEAB) and the corrected standard ellipse area (SEAc) considering 40% of central data points, which is less sensitive to small sample sizes (Jackson et al. 2011). Trophic niche similarity was estimated by calculating the overlap between two ellipses (Jackson and Parnell 2023). The overlap area, measured in per mil squared (‰^2^), was calculated using the SEAc of each ellipse. Trophic niche overlap was used to determine ecological similarity between white‐clawed and signal crayfish populations in allopatry and sympatry. Trophic niche areas of each crayfish population were compared by posterior distribution of paired trophic niches, and their probability of posterior distribution similarity was calculated (Jackson and Parnell 2023). The proportions range from 0 (no overlap) to 1 (complete overlap).

Macroinvertebrate functional groups (collectors, filter feeders, and scrapers) were combined into one food source (macroinvertebrates) due to their similar carbon and nitrogen isotopic values, while macroinvertebrate predators represented a different food source (predators). Juvenile crayfish were also considered a food source for adults (Veselý et al. 2020; Ercoli et al. 2021).

Proportions of six food sources (macroinvertebrates, predator macroinvertebrates, juvenile crayfish, macrophytes, periphyton, and detritus) used by crayfish of both species in allopatry and sympatry were calculated using MixSIAR Bayesian mixing models in R (Stock et al. 2020). MixSIAR models were performed for each site (allopatric and sympatric), using species (white‐clawed and signal crayfish) as fixed factors, and selected residual and process errors (Stock et al. 2020). As recommended by Veselý et al. (2024) and Brauns et al. (2018), fractionation factors for δ^15^N and δ^13^C were assumed to be 2.6‰ ± 2‰ and 0.1‰ ± 2.2‰, respectively, for macroinvertebrates, and 2.4‰ ± 0.42‰ and 0.40‰ ± 0.28‰, respectively, for detritus, macrophytes, and periphyton (McCutchan Jr. et al. 2003). Models were run using Markov Chain Monte Carlo (MCMC) parameters of three chains of 1,000,000 iterations, a burn‐in phase of 500,000, and thinning of 500. Percentage contributions of food sources to crayfish diets were generated as posterior distributions with 95% credibility intervals for each site. Gelman‐Rubin and Geweke tests were used to test convergence and diagnostic statistics of all model results. For the first test, all variables must have values < 1.05, and for the second test, the means of the first and second parts of the chain must be the same. Analyses were performed using the packages *SIBER* (Jackson and Parnell 2023) and *MixSIAR* (Stock et al. 2020) in R ver. 4.1.3 (R Core Team 2022).

### 2.7 Gut Contents

Signal crayfish and white‐clawed crayfish individuals were dissected, and their foreguts were analysed to identify prey items (Ercoli et al. 2021). The contents from each foregut were placed in a Petri dish with a small amount of water and examined under a dissecting microscope (50× magnification). Food items were categorised into macrophytes, macroinvertebrates, indeterminate animals, crayfish remains, periphyton, fine particulate organic matter (FPOM), and detritus (coarse particulate organic matter, CPOM). Identification and counting of macroinvertebrates were conducted by recognising hardened body parts, particularly head capsules, mouthparts, and leg segments (Stewart et al. 2002). For unquantifiable components, such as periphyton and CPOM, visual estimates were made using four abundance classes (ranging from 0 to 3; Bo et al. 2012). The percentage occurrence (%Oi) of food items in both species was determined as:

$$\%O_{i}=\left(J_{i}/P\right)\times 100$$

where *Ji* represents the number of crayfish containing prey item i, and *P* is the total number of crayfish with food in their stomachs.

### 2.8 Body and Physiological Condition

Condition indices were considered to assess potential differences in health and fitness (Peig and Green 2010) between signal crayfish occurring in the sympatric and in allopatric zones. Due to the small sample size collected for white‐clawed crayfish, we could not perform comparisons between signal and white‐clawed crayfish. We used Fulton's Condition Factor (FCF; Fea et al. 2017; Maguire and Klobučar 2011), calculated as: FCF = *W*/TL^3^, where *W* is weight and TL is total length. This index is size‐ and sex‐dependent (Streissl and Hödl 2002); therefore, comparisons were made between individuals of the same sex and adults.

The physiological index as an organ moisture content of hepatopancreas is frequently used to evaluate crayfish condition, their energy reserves, and potential effects of environmental stress (Jussila 1997; Lucić et al. 2012; Rebrina et al. 2015). Each crayfish was weighed and dissected, and the digestive gland (hepatopancreas) was precisely removed and placed in a foil cup. The weight of the wet hepatopancreas was measured using an electronic balance, with an accuracy of 0.001 g. Then, foil cups containing the tissues were dried for 24 h at 80°C and weighed again to determine the dry organ weight. Hepatopancreas moisture content (HM) was calculated using the following formula:

$$\mathrm{HM}=\left(W_{\mathrm{wo}}-W_{\mathrm{do}}\right)\times 100/W_{\mathrm{wo}}$$

where *W*_wo_ represents wet organ weight (g), and *W*_do_ represents dry organ weight (g). The resulting values are inversely proportional to the energetic status (Jussila and Mannonen 1997).

### 2.9 Statistical Analyses

Differences in carbon and nitrogen stable isotopes of signal and white‐clawed crayfish between allopatric and sympatric sites, and differences in body condition (indices) in signal crayfish between sites and sexes were evaluated using the non‐parametric Kruskal–Wallis test. To allow for trophic niche comparisons of white‐clawed and signal crayfish between sites and species, basal resources (predators, periphyton, macrophytes, macroinvertebrates, and detritus) were tested for differences between sites using one‐way ANOVA, which was also employed for identifying differences in macroinvertebrate abundance and species richness (Shannon index) between sites. Significant differences (*p* < 0.05) from ANOVAs were further analysed using a post hoc Tukey‐HSD pairwise test for comparisons between sites. Normality and homogeneity of variances assumptions were tested prior to statistical analyses using Shapiro–Wilk and Levene tests, respectively. All statistical analyses were performed in R (R Core Team 2022).

## 3 Results

In total, 44 white‐clawed crayfish and 134 signal crayfish were collected and analysed for SIA and gut contents (Table 2). At the sympatric site, the sex ratio of white‐clawed crayfish was balanced (M/F = 1.2), but there were more juveniles (*n* = 9) than adults (*n* = 2). In contrast, signal crayfish adults dominated (*n* = 36) compared to juveniles (*n* = 3), and the sex ratio was more unbalanced towards males (M/F = 1.6).

**TABLE 2:** Carbon and nitrogen stable isotope mean values (±standard deviation), cephalothorax length (CL; ±standard deviation), sex, and age class of white‐clawed crayfish (WCC) and signal crayfish (SGC), collected from allopatric sites (V1 and V3) and a sympatric site (V2) in 2017 and 2018.

Year	Site	Species	Age	Sex	N	CL	δ^13^C	δ^15^N
2017	V1	WCC	Adult	F	1	43.9	−27.2	5.14
				M	9	36.0 ± 6.10	−26.4 ± 0.41	4.34 ± 0.27
			Juveniles	F	3	21.7 ± 4.00	−26.7 ± 0.84	4.6 ± 0.63
				M	5	26.6 ± 1.71	−26.7 ± 0.39	4.4 ± 0.18
	V3	SGC	Adult	F	8	45.6 ± 5.48	−27.6 ± 0.7	5.92 ± 0.41
				M	20	46.4 ± 7.37	−27.3 ± 0.67	5.62 ± 0.56
			Juveniles	F	11	15.0 ± 1.80	−26.7 ± 0.60	6.22 ± 0.57
				M	9	16.0 ± 4.89	−27.2 ± 1.00	6.47 ± 0.41
2018	V1	WCC	Adult	F	3	35.0 ± 1.22	−26.9 ± 0.13	4.42 ± 0.26
				M	3	37.0 ± 7.01	−27.1 ± 0.26	4.58 ± 0.33
			Juveniles	F	4	23.5 ± 2.68	−26.9 ± 0.64	3.87 ± 0.36
				M	5	23.8 ± 3.52	−26.7 ± 0.5	4.02 ± 0.43
	V2	WCC	Adult	F	1	34.8	−26.4	4.94
				M	1	30.4	−27.2	4.59
			Juveniles	F	4	22.9 ± 1.39	−27.8 ± 0.53	4.38 ± 0.06
				M	5	24.6 ± 1.86	−27.3 ± 0.64	4.68 ± 0.41
		SGC	Adult	F	13	40.3 ± 6.82	−27.6 ± 0.68	4.25 ± 1.11
				M	23	38.7 ± 6.75	−27.5 ± 0.66	3.96 ± 0.89
			Juveniles	F	2	24.2 ± 7.26	−27.8 ± 0.14	3.67 ± 0.10
				M	1	18.9	−28.6	3.47
	V3	SGC	Adult	F	21	46.2 ± 8.05	−27.9 ± 0.46	6.36 ± 0.50
				M	18	48.4 ± 8.13	−28.1 ± 0.50	5.87 ± 0.55
			Juveniles	F	6	28.3 ± 1.84	−28.1 ± 0.38	6.3 ± 0.13
				M	2	24.9 ± 6.95	−27.1 ± 1.70	6.41 ± 0.46

### 3.1 Aphanomyces astaci

Molecular analyses performed on a total of 42 signal crayfish and 32 white‐clawed crayfish did not detect the presence of *A. astaci* . These findings align with the absence of mass mortality events, which have not been recorded since the evidence of the invasive crayfish in Valla Stream. Additionally, no mortality events associated with crayfish plague were observed during or after this study (respectively 2017–2018 or 2021).

### 3.2 Macroinvertebrates

**FIGURE 3:**
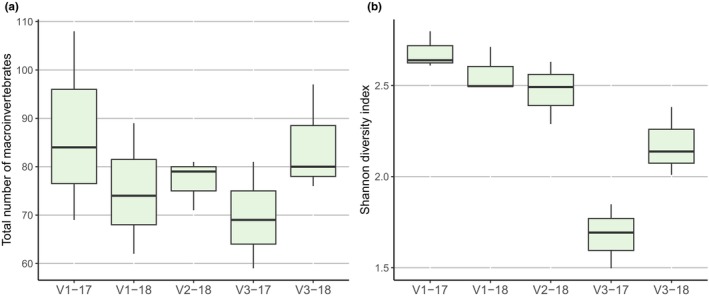
Abundance (a) and Shannon diversity index (b) of macroinvertebrates at the allopatric white‐clawed crayfish site (V1), white‐clawed and signal crayfish sympatric site (V2), and the allopatric signal crayfish site (V3) in 2017 and 2018. Horizontal black lines within the box represent the median; lower and upper areas within the box, lower and upper limits of the box, and error bars represent credible intervals of 50%, 75%, and 95%, respectively.

Total macroinvertebrate abundance did not significantly differ among sites in both years (*F* = 0.88, df = 4, *p* = 0.51), but the Shannon diversity index varied significantly between sites (*F* = 19.89, df = 4, *p* < 0.001) (Figure 3). In 2017, macroinvertebrate diversity at the allopatric site (V3) significantly differed from all other sampling sites (*p* < 0.02). In 2018, diversity decreased from V1 to V3, though the statistical significance was marginal (*p* = 0.07) (Figure 3).

### 3.3 Trophic Niches

Carbon stable isotopes in signal crayfish tissue did not differ between allopatric (V3) and sympatric (V2) sites (*p* = 0.61), but nitrogen isotopes were significantly (*p* < 0.001) higher at the allopatric site (V3). White‐clawed crayfish indicated lower carbon isotope values at the sympatric site (*p* = 0.003).

**FIGURE 4:**
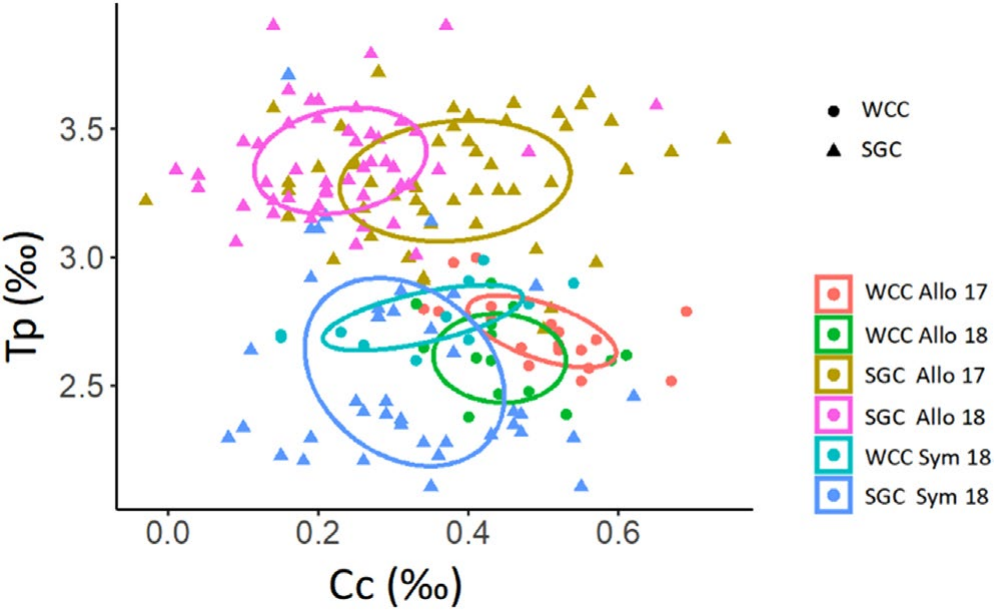
Trophic niches of signal (SGC) and white‐clawed (WCC) crayfish in sympatric (2018) and allopatric (2017 and 2018) sites. $\mathrm{Tp}\;\left(\text{Trophic position}\right)=\delta^{15}N_{\mathrm{cor}.}\;\mathrm{Cc}=\delta^{13}C_{\mathrm{cor}.}$

SIBER models revealed that white‐clawed crayfish shifted from enriched carbon‐corrected values in allopatry to more depleted values in sympatry, while signal crayfish shifted from a higher trophic niche at allopatric sites to a lower one at the sympatric site (Figure 4). Both species altered their trophic niches, transitioning from non‐overlapping niches in allopatry to markedly overlapping (94% of the WCC and 34% of the SGC) in sympatry (Figure 4).

**FIGURE 5:**
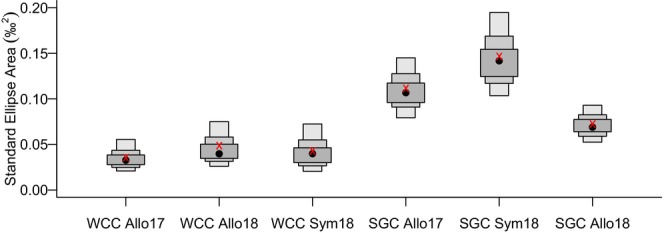
Box plots indicate Bayesian Standard Ellipse Area (SEA.B) for white‐clawed (WCC) and signal (SGC) crayfish in allopatry (2017 and 2018) and sympatry (2018). Black dots, darker grey, dark grey and light grey represent median, 50%, 75%, and 95% of Credibility Interval. Red crosses indicate the corrected Standard Ellipse Area SEAc.

At allopatric sites, signal and white‐clawed crayfish exhibited trophic niche partitioning, with signal crayfish occupying a higher trophic level than white‐clawed crayfish (Figure 4). However, within the same species, trophic niches partially overlapped between 2017 and 2018 (35% for WCC and 34% for SGC; Figure 4). The trophic niche areas of signal crayfish were wider than those of white‐clawed crayfish and particularly wide at sympatric sites (0.12‰) compared to allopatric sites in 2017 (0.08‰) and 2018 (0.05‰; Figure 5). In contrast, white‐clawed crayfish maintained a consistent trophic niche area across years and sites (Figure 5).

### 3.4 MixSIAR

**FIGURE 6:**
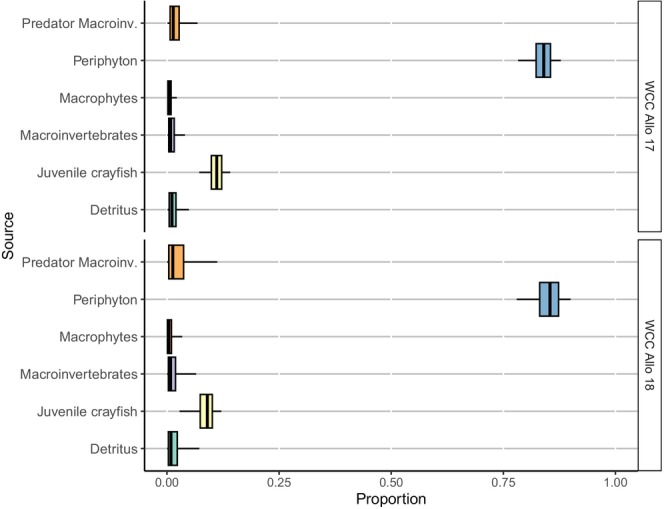
Proportions of the food sources in the diet of white‐clawed crayfish (WCC) at the allopatric site (V1) in 2017 and 2018 estimated from the Bayesian MixSIAR model output. Black lines represent the median; lower and upper areas of the box, lower and upper limits of the box, and error bars represent credible intervals of 50%, 75%, and 95%, respectively.

**FIGURE 7:**
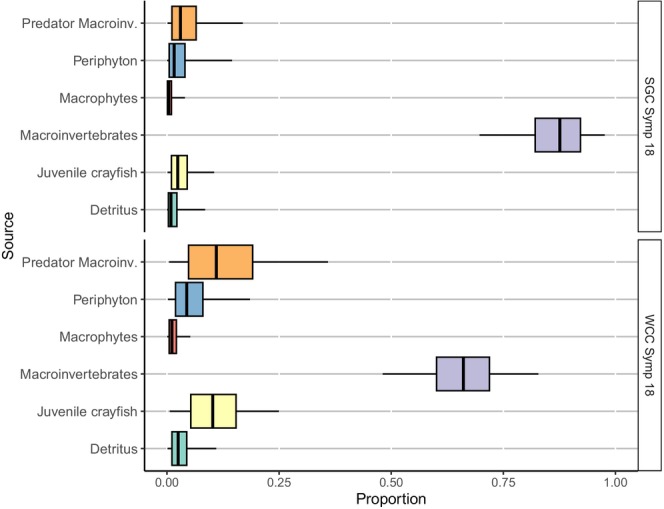
Proportions of the food sources in the diet of white‐clawed (WCC) and signal (SGC) crayfish at the sympatric site (V2) in 2018 estimated from the Bayesian MixSIAR model output. Black lines represent the median; lower and upper areas of the box represent lower and upper limits of the box; and error bars represent credible intervals of 50%, 75%, and 95%, respectively.

**FIGURE 8:**
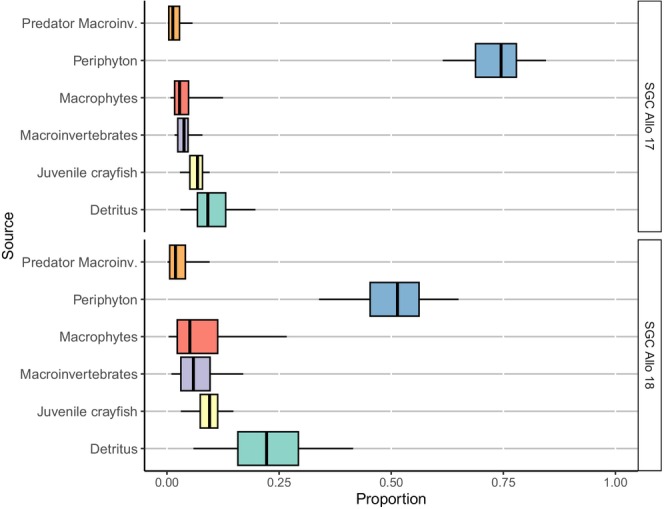
Proportions of the food sources in the diet of signal crayfish (SGC) at the allopatric site (V3) in 2017 and 2018 estimated from the Bayesian MixSIAR model output. Black lines represent the median; lower and upper areas of the box represent lower and upper limits of the box; and error bars represent credible intervals of 50%, 75%, and 95%, respectively.

As with the SIBER results, MixSIAR mixing models indicated that the diets of both white‐clawed and signal crayfish did not vary between years in allopatry, with both species primarily consuming periphyton, ranging from 52% to 85% (Figures 6 and 8). White‐clawed crayfish mostly consumed periphyton in allopatry (84% in 2017 and 85% in 2018), but shifted to macroinvertebrates (66%) when coexisting with signal crayfish (Figures 6 and 7). In contrast, signal crayfish in allopatry relied on a mix of periphyton (74% in 2017 and 52% in 2018), detritus (9% in 2017 and 22% in 2018), and juvenile conspecifics (7% in 2017 and 10% in 2018), but shifted to macroinvertebrates (88%) when in sympatry with white‐clawed crayfish (Figures 7 and 8).

Interestingly, signal crayfish exhibited more cannibalistic behaviour in allopatry (7% to 10%) than in sympatry (2%), possibly due to higher population density in allopatry (Figures 7 and 8). In contrast, white‐clawed crayfish displayed consistent cannibalistic behaviour at both allopatric (9% to 11%) and sympatric (10%) sites (Figures 6 and 7).

### 3.5 Gut Contents

**FIGURE 9:**
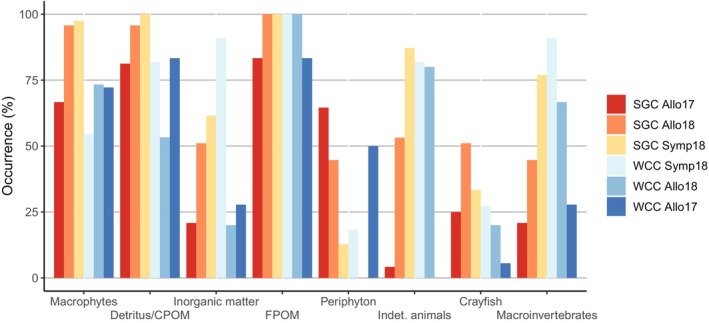
Food item occurrence in stomachs of signal (SGC) and white‐clawed (WCC) crayfish in Valla Stream occurring in allopatry (Allo17, Allo18) and sympatry (Symp18).

Gut content analysis results generally aligned with Bayesian model results. However, model results indicated that macrophytes and detritus were not part of the diets of the two crayfish species, while a high occurrence (more than 50%) of FPOM, CPOM, and macrophytes was found in the stomachs of both crayfish species across all sites (Figures 6, 7, 8, 9). Periphyton was consumed more when white‐clawed crayfish (50% in 2017) and signal crayfish (65% in 2017 and 45% in 2018) were alone. In contrast, less periphyton (13% for signal crayfish and 18% for white‐clawed crayfish) and significantly more macroinvertebrates (77% for signal crayfish and 91% for white‐clawed crayfish) were consumed when the species were coexisting (Figure 9). This pattern aligns with stable isotope results, which showed that the proportion of periphyton increased and macroinvertebrates decreased when the two crayfish species lived in allopatry, and vice versa when the species co‐occurred (Figures 6, 7, 8).

Although crayfish remains were found in the stomachs of both crayfish species, cannibalistic behaviour was higher in signal crayfish (51% in 2018) than in white‐clawed crayfish (5% in 2017; Figure 9).

### 3.6 Condition Indices

**FIGURE 10:**
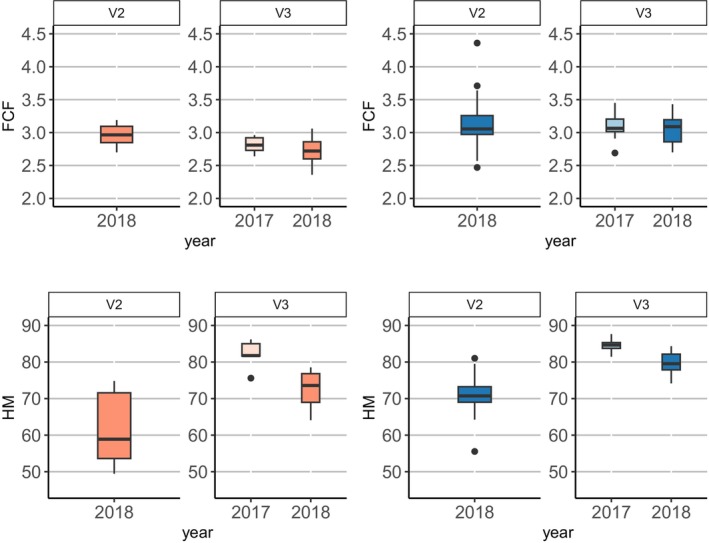
Boxplots represent the Fulton (FCF) and hepatopancreas (HM) indices of signal crayfish females (pinks) and males (blues) at the invasion front (sympatry; V2) and allopatric (V3) sites.

Comparisons of indices were limited to signal crayfish samples due to the small sample size of white‐clawed crayfish. In signal crayfish females, both indices differed among sites (FCF, *χ* ^2^ = 7.82, df = 2, *p* = 0.020; HM, *χ* ^2^ = 15.69, df = 2, *p* < 0.001), while male individuals showed differences only in HM (FCF, *χ* ^2^ = 0.06, df = 2, *p* = 0.97; HM, *χ* ^2^ = 24.82, df = 2, *p* < 0.001; Figure 10). In 2018, signal crayfish females from the sympatric site (V2) showed better body (FCF, *p* = 0.024) and physiological (HM, *p* = 0.021) conditions compared to females at the allopatric site (V3), as well as males for physiological condition (HM, *p* < 0.001; Figure 10).

## 4 Discussion

This study investigated and compared the trophic interactions between invasive signal crayfish and native white‐clawed crayfish at sites of co‐occurrence and in allopatry. The presence of crayfish plague, body condition, and trophic niche traits were explored as possible factors that make invasive signal crayfish a strong competitor, enhancing its expansion and replacing the native white‐clawed crayfish.

In the Valla Stream, invasive crayfish have been established for over a decade (Candiotto et al. 2010) and are progressively displacing the native crayfish population. Supporting our hypothesis, tests for *A. astaci* were negative for all specimens collected, including individuals in sympatry (V2), where both species were found to co‐occur. Recent studies (Rusch et al. 2020) suggest that the co‐occurrence of signal crayfish and native crayfish may only be possible in the absence of crayfish plague. However, the signal crayfish invasion in Valla Stream is progressing, outcompeting the native white‐clawed crayfish. Therefore, the progressive replacement of the native species may be driven by factors other than crayfish plague. Sub‐lethal impacts could facilitate competitive exclusion, playing an important role in interactions between white‐clawed crayfish and signal crayfish (Haddaway et al. 2012). The presence of microsporidia was evaluated in the white‐clawed crayfish population in Valla Stream (Fea et al. 2024). Evidence of *Astathelohania contejeani* was confirmed by molecular tests in three specimens plus one co‐infected by both pathogens (*A. contejeani* and *Nosema austropotamobii*) over 130 individuals macroscopically evaluated for signs of microsporidia (i.e., whitish abdomen). Low prevalence levels of microsporidia infections do not appear to affect the overall fitness of native crayfish populations, as they typically progress slowly as chronic infections. Therefore, we can exclude microsporidia as a significant factor contributing to the decline of white‐clawed crayfish in the Valla Stream, as well as the presence of Branchiobdellids (Mazza et al. 2019) is expected to have minimal impact on competitive interactions between native and invasive crayfish species (Anderson et al. 2021).

Dynamics of the crayfish disease may be linked to poor water quality, as a significant relationship was found between the chemical status of the water body and the prevalence of *A. astaci* , but not for *T*. *contejeani* nor bacilliform virus (Alvanou et al. 2024; Anderson et al. 2021). The prevalence of *A. astaci* was higher in catchments classified as failing in terms of chemical status (Anderson et al. 2021). Unfortunately, the Valla Stream catchment is not included in the network of measurement stations within the Water Framework Directive (2000/60/EC). However, based on our macroinvertebrate dataset and the measured chemical and physical parameters (see Table S1 in Supporting Information), the water quality of Valla Stream is high, which may explain the no detection of *A. astaci* .

Our results align with previous studies (Ercoli et al. 2014; Olsson et al. 2009), which found that signal crayfish consistently exhibit wider trophic niches and likely greater trophic flexibility compared to native crayfish. The broader trophic niche of signal crayfish suggests an omnivorous diet, deriving energy from diverse sources, unlike the more conservative trophic niche of white‐clawed crayfish. However, contrary to Jackson et al. (2016), signal crayfish in Valla Stream exhibited a wider trophic niche in sympatry than in allopatry. This result suggests that co‐occurrence forces the invasive crayfish species to adopt a more generalist diet, exploiting a broader range of food resources or habitats, which highlights its high degree of trophic plasticity. As hypothesized, signal crayfish shifted to a lower trophic level and expanded their trophic niche in sympatry, indicating higher adaptability and competitiveness compared to the more conservative native white‐clawed crayfish. Trophic niche shift towards lower or intermediate trophic levels was also observed in invasive fish species (Comte et al. 2017). However, in Valla Stream, a shift to a lower trophic level occurred when in sympatry, while in allopatry, the invasive species occupied the higher trophic level. In contrast with Tran et al. (2015), invasive signal and native white‐clawed crayfish occupied similar trophic niches only when co‐occurring. In allopatry, the two species indicated trophic partitioning. Moreover, when co‐occurring, signal crayfish occupied most of the trophic niche of white‐clawed crayfish, while the latter occupied a much smaller area of the signal crayfish trophic niche, indicating stronger interspecific competition for the native species. Previous studies also found wider and higher overlap of trophic niches by signal crayfish when compared with native counterparts (Ercoli et al. 2014; Olsson et al. 2009), but the comparisons were not assessed in sympatry. As suggested by Pacioglu et al. (2020), successful coexistence between native and invasive crayfish species could only occur if there is trophic partitioning between the two species. In our study, the invasive and native species showed significant trophic niche overlap under sympatric conditions, making their stable coexistence unlikely. The progressive replacement of native crayfish by invasive ones, observed in the years following our study, could confirm this co‐occurrence as a frame at the invasion front during the slow invasion process.

Our results indicate that CPUE values at the sympatric site (V2) were low for both species, particularly for signal crayfish. It is worth noting that, according to the regional permit previously obtained, we were only allowed to collect a small sample size of native white‐clawed crayfish. A decrease in conspecific density at the invasion front of a population is an expected consequence of dispersal (Phillips 2016). A sympatric situation between narrow clawed crayfish ( *Astacus leptodactylus* ) and signal crayfish in a Croatian river revealed only a few individuals of the native species and only 3.3% of the invasive one at the invasion front (Hudina et al. 2012; Rebrina et al. 2015). The abundance of signal crayfish males at the sympatric site in October 2018 suggests a potential bias towards male dispersal during the initial spread of invasive signal crayfish. Male‐biased sex ratios at the invasion front have been observed for signal crayfish (Hudina et al. 2012; Rebrina et al. 2015). Crayfish males often exhibit more aggression and competition than females for resources, including food, shelter, and mates (Fero and Moore 2008). Consequently, they may gain an advantage from dispersing to zones with lower densities of conspecifics to avoid interspecific competition. The intensity of aggression depends on crayfish size, and the most aggressive interactions are usually recorded in the largest individuals (Moore 2007). When both native crayfish and crabs were present in the co‐occurrence area, crustacean size was slightly smaller than crayfish and crab sizes at their allopatric sites (Mazza et al. 2017). Abiotic factors such as temperature and adverse climatic events can influence the distribution of invasive species, directly or indirectly affecting their dispersal, establishment, and impact on native communities (Capinha et al. 2013; Rodríguez Valido et al. 2021). In a recent study, Ghia, Fea, et al. (2024) investigated the effects of drought conditions on the dispersal behaviour of signal crayfish and red swamp crayfish ( *Procambarus clarkii* ). The study highlighted the strong ability of signal crayfish to move towards more favourable environmental conditions. In Valla stream, drought conditions are common during summer and can pose significant stress on native communities, including white‐clawed crayfish, while affecting the more resilient invasive signal crayfish to a lesser extent.

Invasive crayfish diets, trophic niche, and body condition in invaded ecosystems may depend on their abundance (Almeida et al. 2013; Jackson et al. 2017) or be influenced by environmental conditions such as differences in habitats and food source availability between allopatric and sympatric sites (Jackson and Britton 2014; Pacioglu et al. 2020). In Valla Stream, the three sites indicate similar habitat and food resources availability (macroinvertebrates abundance), suggesting that shifts in diet and trophic niches were driven by ecological traits of the crayfish species rather than by environmental variables. Moreover, signal crayfish were less abundant in sympatry than in allopatry. Lower population density at the invasion front could lead to greater food resource availability for individuals, resulting in a broader trophic niche. Results from MixSIAR models and stomach contents of both species indicated a more protein‐based diet when co‐occurring and a more plant‐based diet when in allopatry, suggesting that both species needed a protein‐rich diet in sympatry, regardless of their age class. For the native white‐clawed crayfish, this diet may be a result of coping with interspecific competition. In contrast, for signal crayfish, the protein‐based diet could be linked to higher energy demand and investment required in sympatric sites, potentially as a strategy to support invasion. Previous work showed that signal crayfish had better body condition and energetic status, especially in females, in newly invaded habitats (Rebrina et al. 2015). Jackson and Britton (2014) found that sympatric invasive crayfish species generally exert either additive or amplified effects at community and ecosystem levels through trophic cascade effects. In our study, although the density of crayfish species recorded in sympatry was low, their substantial trophic niche overlap and protein‐based diet could suggest strong negative impacts on the macroinvertebrate community, potentially causing detrimental effects on the stream ecosystem via cascade effects.

In our study, Fulton and hepatopancreas index analyses revealed that both female and male signal crayfish were in better physiological condition in sympatry than in allopatry. These results support the hypothesis that improved body condition of signal crayfish at the invasion front could be a critical factor in invasion success. Our results align with those of Rebrina et al. (2015), which found that both male and female signal crayfish were stronger and healthier at the invasion front compared to the core site. Unfortunately, the small sample size in our study prevented hepatopancreas and Fulton index analyses of white‐clawed crayfish for comparison with signal crayfish at the invasion front. We acknowledge that the limitations of our study, including the spatial (a single stream studied) and temporal (a 2‐year sampling period) constraints, may restrict the broader applicability of our findings. Limited resources and the rare coexistence of the two species in nature prevented us from studying their ecological traits on larger temporal and spatial scales. However, this rare coexistence provided a unique opportunity to better understand the ecological relationships between the two crayfish species in the context of invasion dynamics. Nonetheless, more research studies integrating invasion and behavioural ecology with genetic traits would help to fill the knowledge gap on the mechanisms and strategies that make an invasive species successful (Mueller et al. 2017).

In conclusion, the native, white‐clawed crayfish population in Valla Stream is experiencing strong detrimental effects from signal crayfish invasion, which is steadily displacing the native species, even though crayfish plague was not detected. The fate of native crayfish in Valla Stream mirrors that of other native species studied in co‐occurrence with invasive crayfish, facing intense interspecific competition due to trophic niche overlap. This competition has led to a retraction of the native population towards headwater streams, while invasive crayfish continue to expand their range (Chucholl 2016; Viana et al. 2023; Oficialdegui et al. 2024). This study provides further evidence of the urgent need for immediate action to stop or mitigate the upstream invasion of signal crayfish, such as through eradication efforts and the installation of physical barriers. A new method for controlling invasive crayfish spreading is currently under study in laboratory experiments (Ghia, Fea, et al. 2024; Ghia, Morabito, et al. 2024) and in mesocosms (Ghia et al. under review) with the intent to implement the method in nature in the near future. Additionally, protecting the already endangered white‐clawed crayfish in Valla Stream should involve reintroduction programs.

## Author Contributions

**Daniela Ghia:** conceptualization (equal), formal analysis (equal), investigation (equal), writing – original draft (supporting). **Gianluca Fea:** investigation (equal), writing – original draft (supporting). **Annagiulia Murtas:** formal analysis (supporting), investigation (equal). **Martina Ventimiglia:** formal analysis (supporting), investigation (equal). **Tiziano Bo:** formal analysis (supporting), investigation (equal). **Andrea Basso:** formal analysis (equal), writing – original draft (equal), writing – review and editing (equal). **Tobia Pretto:** formal analysis (equal), writing – original draft (supporting). **Roberto Sacchi:** supervision (equal), writing – original draft (supporting). **Fabio Ercoli:** conceptualization (equal), formal analysis (lead), supervision (equal), writing – original draft (lead).

## Conflicts of Interest

The authors declare no conflicts of interest.

## Supporting information


Data S1

## Data Availability

The datasets analysed during the current study are uploaded as Data S1.
