# Supplementary material for: Better Alone Than in Bad Company: Trophic Ecology of Co‐Occurring Invasive and Native Crayfish
Source: Ecol Evol. 2025 May 14;15(5):e71385. doi: 10.1002/ece3.71385 (PMC12076067; doi:10.1002/ece3.71385)
Supplement: Supplementary file 1 — Data S1 [file ECE3-15-e71385-s001.zip › Table S1.docx]

| Year/Site | Conductivity (μS/cm) | Water Temp (°C) | pH | O_2_  (%) | O_2_ (mg/L) | CaCO_3_ (mg/L) | NO_2_ (mg/L) | NO_3_ (mg/L) | PO_4_ (mg/L) | SO_4_ (mg/L) | Cl_2_ (mg/L) | Fe (mg/L) | Cu (mg/L) | NH_4_ (mg/L) |
| --- | --- | --- | --- | --- | --- | --- | --- | --- | --- | --- | --- | --- | --- | --- |
| 2017 – V1 | 233 | 13.3 | 7.9 | 87.8 | 8.67 | 138 | 0.010 | 5.7 | 0.17 | 7 | 0.08 | 0.02 | 0.06 | 0.00 |
| 2017 – V2 | 318 | 14.4 | 7.8 | 71 | 7.08 | 168 | 0.009 | 6.2 | 0.11 | 10 | 0.05 | 0.02 | 0.01 | 0.00 |
| 2017 – V3 | 342 | 16.3 | 8.2 | 52.5 | 5.08 | 168 | 0.053 | 6.5 | 0.22 | 0 | 0.14 | 0.04 | 0.00 | 0.04 |
| 2018 – V1 | 215 | 12.5 | 7.6 | 89 | 9.17 | 115 | 0.022 | 8.2 | 0.20 | 4 | 0.14 | 0.01 | 0.02 | 0.00 |
| 2018 – V2 | 274 | 13.6 | 7.6 | 71.5 | 7.08 | 153 | 0.017 | 6.8 | 0.21 | 4 | 0.08 | 0.02 | 0.03 | 0.00 |
| 2018 – V3 | 246 | 15 | 8.4 | 106.7 | 10.5 | 161 | 0.015 | 3.4 | 0.25 | 1 | 0.02 | 0.01 | 0.01 | 0.00 |
